# A retrospective cohort study on 3D printed temporary crowns

**DOI:** 10.1038/s41598-024-68354-2

**Published:** 2024-07-27

**Authors:** Michael del Hougne, Isabella Di Lorenzo, Christian Höhne, Marc Schmitter

**Affiliations:** Department of Prosthodontics, University of Würzburg, Pleicherwall 2, 97070 Würzburg, Germany

**Keywords:** Crown, Survival, 3D print, CAD/CAM, Additive manufacturing, Dental, Dentistry, Prosthetic dentistry, Fixed prosthodontics

## Abstract

In this retrospective cohort study the survival rate, clinical parameters, patient satisfaction with aesthetics and oral health-related quality of life of 3D printed temporary crowns were evaluated. Temporary crowns were 3D printed with a Form3B out of Permanent Crown Resin (Formlabs GmbH). Anonymized data for the restorations’ survival (in-situ) was evaluated retrospectively for 98 temporary crowns of 63 patients fabricated within 19 months. Among these restorations, further analysis was conducted for 42 temporary crowns of 24 patients regarding clinical parameters (modified USPHS criteria), patient satisfaction with aesthetics and impact on oral health-related quality of life (OHRQoL) (OHIP 14). Descriptive statistical analysis (significance level of α = 0.05) included a Kaplan–Meier curve for survival analysis, a Kolmogorov–Smirnov test for USPHS, aesthetics and OHIP data, followed by a Wilcoxon test for USPHS and OHIP data and Chi-squared test for aesthetics data. Cronbach’s alpha was calculated for OHIP data. The average observation period for survival analysis was 256 days. The survival rate was satisfactory at 98% and n = 2 catastrophic failures (i.e. fracture) occurred. Total OHIP scores, with good reliabilities, improved from 6.63 to 2.21 significantly (*p* = 0.005) and patient satisfaction with aesthetics (*p* < 0.001) as well. Clinical analysis with modified USPHS criteria revealed encouraging results.

## Introduction

Crowns are an established restorative method with the availability of various restorative materials. Due to computer assistance, computer-aided-design (CAD) / computer-aided-manufacturing (CAM) represents a development that enables further options^1^. CAM includes subtractive (s-CAM) and additive (a-CAM) manufacturing of materials such as ceramics, alloys or resins. S-CAM is considered the gold standard within its limitations^2^.

3D printing, representing an a-CAM method, is a novel production method and can be utilized to produce resin-based products such as crowns, bridges, bite splints or prothesis. While some materials enable a permanent intraoral use or restoration, other materials are approved for a temporal intraoral use only. Generally, materials require an approvement as legitimated for intraoral use. Overall, there is an increasing popularity of 3D printing in dentistry, however, limited information on clinical performance is available^3^. Clinical outcomes of resin-based crowns fabricated with s-CAM have been reported^4^ and survival rates of 100% after 6 months^5^ and 87.9% after 3 years^6^ were observed. However, limited data is available for the survival rate and clinical performance of a-CAM restorations.

Permanent Crown Resin (Formlabs GmbH, Berlin, Germany) is a material for additive manufacturing of 3D printed biocompatible crowns for permanent use. It can be utilized to produce single crowns, onlays, inlays and veneers with unlimited residual duration. Formlabs states that Permanent Crown Resin restorations are accurate with precise fitment and low tendency of aging effects. Temporary crowns are utilized for teeth with unclear prognosis, such as severe attachment loss or defects, and represent a crucial step in pre-prosthetic treatments.

The aims of this retrospective cohort study were to evaluate the survival of 3D printed temporary crowns and analyze their clinical parameters, satisfaction with aesthetics and impact on oral health-related quality of life (OHRQoL).

The null hypothesis was that 3D printed temporary crowns fabricated out of Permanent Crown Resin perform poorly compared to resin-based s-CAM restorations (data obtained from literature) regarding their technical survival.

## Materials and methods

This retrospective cohort study has been approved by the Institutional Review Board (University of Würzburg, Germany) and usage of anonymized data of the Department of Prosthodontics of the University of Würzburg for the observation period from 01 December 2021 to 30 June 2023 was granted on 11 July 2023 (20230710 01). All methods were performed in accordance with the named guidelines, regulations and Declaration of Helsinki.

### Study design

Patients from the Department of Prosthodontics of the University of Würzburg with 3D printed temporary crowns out of Permanent Crown Resin between 01 December 2021 and 30 June 2023 were included in this study – overall 63 patients with a total of 98 temporary crowns. All types of teeth were included. There were no exclusion criteria upon tooth location, endodontic status, attachment level or patients’ temporomandibular disorder and bruxism. A prosthetic preparation for a crown with a chamfer type of finish line was performed on all teeth, under the premise of a defect oriented preparation, i.e., surpassing build-up fillings where existing.

Survival was defined by the in-situ criterion. A catastrophic failure was defined by a technical defect, i.e., chipping or fracture, which required a new restoration. All other complications were, if documented, recorded, e.g., biological complication of the tooth, removal of the temporary crown for replacement with a permanent crown or cementation failure.

Among the 98 temporary crowns, further analysis was conducted for 42 temporary crowns of 24 patients. These 42 restorations were provided in predoctoral courses and reevaluated at recalls, supervised by experienced dentists under a standardized protocol. Within the predoctoral courses a thorough documentation was conducted, enabling the further analysis for these 42 restorations. Analysis of clinical parameters was enabled via modified USPHS criteria (Table 1), providing descriptives for an evaluation with “alpha”, “bravo”, “charlie” or “delta”. Patient satisfaction with aesthetics and OHRQoL were recorded at baseline before treatment and at recall sessions. For estimating patient satisfaction with aesthetics, the Wong-Baker FACES scale was utilized, a scale with six faces spanning from a happy face to a crying face. This scale is often used for self-assessment and communication of severity of pain^7^. In this study it was utilized to compare satisfaction with aesthetics of the corresponding area before treatment and the temporary crown in the recall session. OHRQoL was determined via the shortened version of the Oral Health Impact Profile (OHIP 14) in its German version (OHIP-G 14), providing a Lickert scale with numerical values where higher sum values represent a compromised OHRQoL and vice versa.

**Table 1:** Modified USHPS criteria for evaluation of clinical parameters.

	Alpha	Bravo	Charlie	Delta
Surface structure	Sound	Rough	–	–
Anatomical form	Sound	Loss of printable material	Loss of material extending to the tooth surface	Complete or partial (> 50%) loss of the restoration
Marginal integrity	Sound	Positive / negative step, removable by finishing	Negative step, not removable by finishing	Strong negative step, not removable in major parts
Marginal discoloration	None	Slight discoloration, removable by finishing	Discoloration, localized, not removable	Strong discoloration in many parts, not removable
Secondary caries	None	Caries present	–	–
Marginal inflammation	None, no pockets > 3mm, no bleeding	Slight, no pockets > 3mm, bleeding	Moderate, pockets < 4-5mm, bleeding	Severe, pockets ≥ 6mm, bleeding
Color stability	No change	Change of color in comparison to baseline	–	–
Color match	Sound	Imperceptible at talking distance	Perceptible at talking distance	Total mismatch
Postoperative sensitivity to air	None	Moderate	Severe	–

### 3D printed temporary crowns

**Figure 1:**
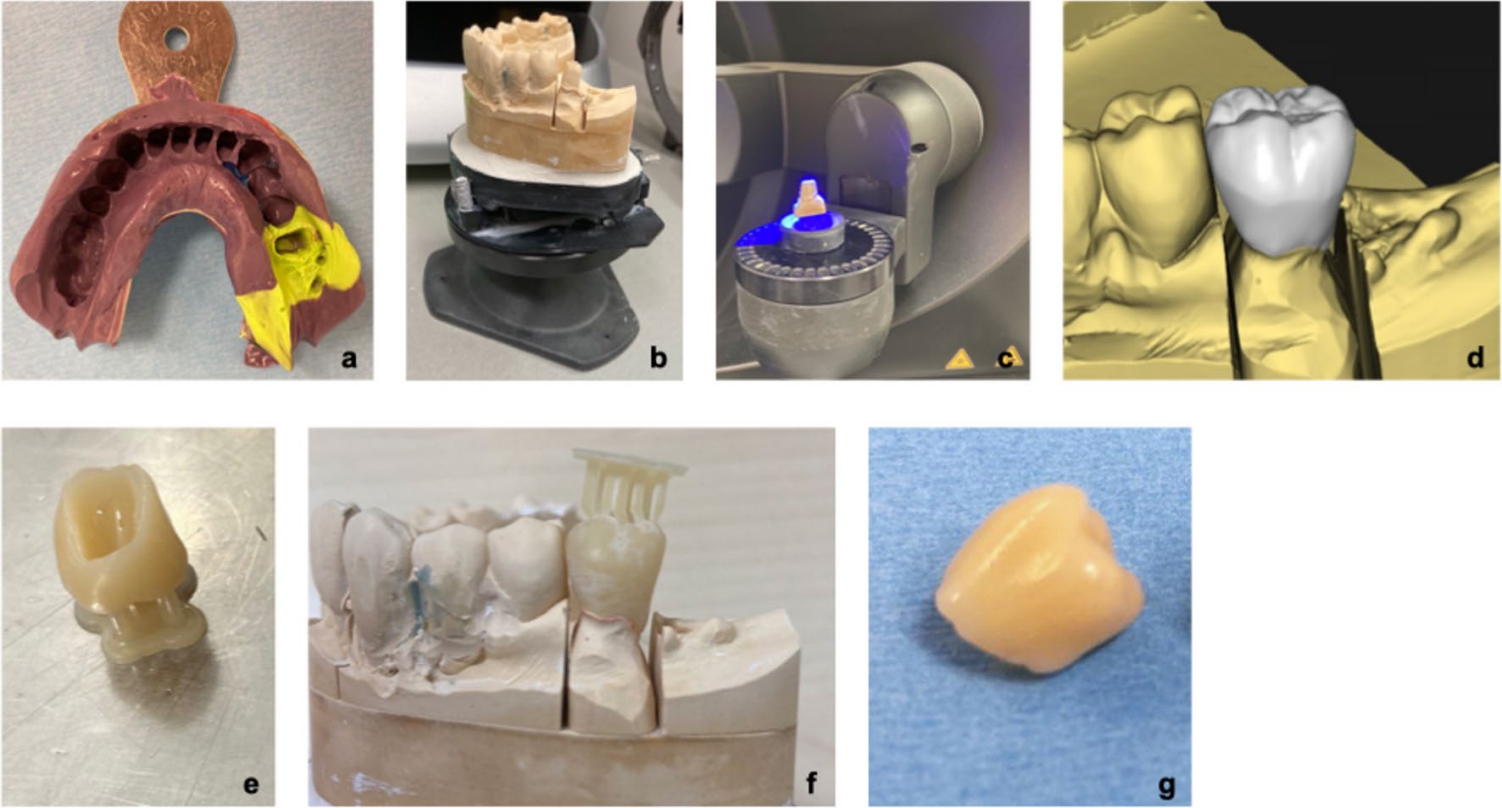
Exemplary production process of a 3D printed temporary crown. (Source: own figure) (**a**). Conventional impression (**b**). Plaster model (**c**). Digitalization with a laboratory scanner (**d**). Designed restoration in a virtual model (**e**). 3D printed restoration with resin residues and support structures (**f**). Test fitting of non-finished restoration on the plaster model (**g**). Finished 3D printed temporary crown.

Fabrication (see Fig. 1 for an exemplary production process) of all restorations followed a standardized protocol: After the preparation of the teeth, followed by conventional impression taking, plaster models were cast and digitalized with the laboratory scanner inEos X5 (Dentsply Sirona, Bensheim, Germany). Restorations were designed in accordance with Formlabs’ guidelines utilizing inLab 20 (Dentsply Sirona, Bensheim, Germany). The general construction requirements resulted in a minimum thickness of the restorations of 1mm, the cement gap was set at 0.04 mm and the drill compensation was disabled. The restorations were imported into the printing software Preform (Formlabs GmbH, Berlin, Germany) for upload to a Form3B (Formlabs GmbH, Berlin, Germany). Temporary crowns were 3D printed with Permanent Crown Resin. Postprocessing was conducted in accordance with the manufacturer’s guidelines by washing, drying, removing supports, post-curing, blasting, finishing, and polishing the temporary crowns. All restorations were cemented with RelyX Unicem (3M ESPE AG, Seefeld, Germany).

### Statistics

A minimal sample size of n = 66 restorations was calculated for a relevant statement upon the survival rate, assisted by the Department of Clinical Epidemiology and Biostatistics of the University of Würzburg, under the assumptions that a good survival rate is represented by a failure rate per year of equal or less than 2% and a bad survival rate is represented by 15% or above. As 98 restorations were fabricated between 01 December 2021 and 30 June 2023, all were included in this retrospective cohort study.

Anonymized data was evaluated and analyzed, utilizing Excel (Microsoft Cooperation, Redmond, USA) and IBM SPSS Statistics (Version 29; IBM, Armonk, USA). Descriptive analysis was conducted, and the significance level was set at α = 0.05. For survival analysis, a Kaplan–Meier curve was generated. Kolmogorov–Smirnov was utilized for testing the normality of the distribution of USPHS, aesthetics and OHIP data, followed by the Wilcoxon test for OHIP data and the Chi-squared test for aesthetics data. Cronbach’s alpha was calculated for OHIP data to determine the reliability.

## Results

### Survival

98 temporary crowns of 63 patients were included with a mean observation time (standard deviation) of 266 (± 130) days, reaching from 18 to 557 days (Table 2). Patients were 63 years old on average and included 27 men and 36 women.

**Table 2:** Observation periods for survival analysis and further analysis (days).

	Survival analysis	Further analysis
Mean	255.99	210.43
Median	244.50	190.00
Standard deviation	129.77	120.49
Minimum	18	29
Maximum	557	535

**Figure 2:**
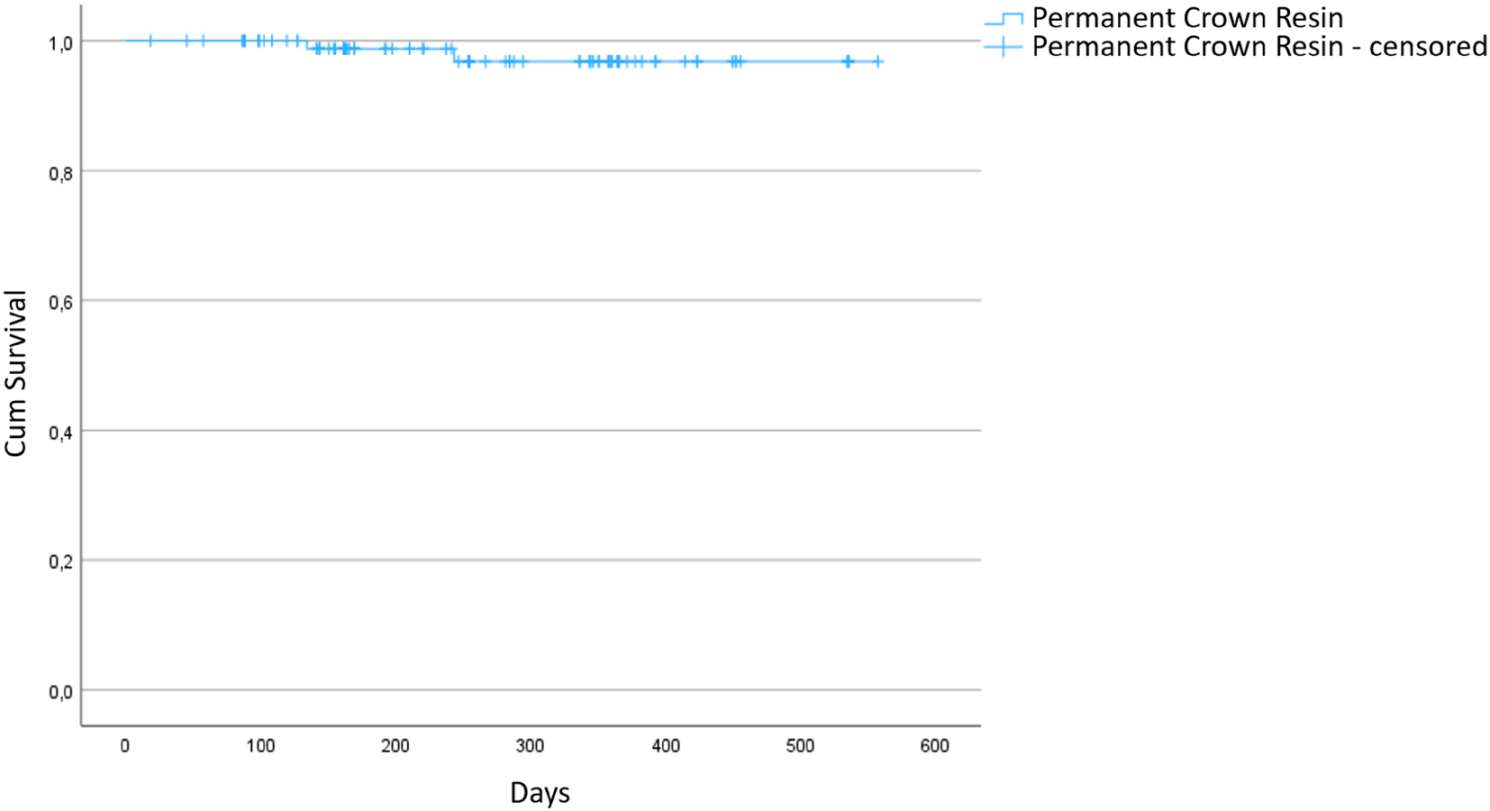
Kaplan–Meier curve of temporary 3D printed crowns. (Source: own figure).

Within the observation period two catastrophic failures occurred due to chipping and fracture resulting in 96 censored restorations. Other non-catastrophic complications included decementation (n = 2), loss of teeth due to trauma and endodontic complication (n = 2), and removals for replacement with a permanent crown (n = 7) – see Table 3. Data was visualized with a Kaplan–Meier curve (Fig. 2).

**Table 3:** Complications with counts and time spans.

Complication	Count	Time span till event (days)
Fracture (catastrophic failure)	2	243
		134
Decementation	6	392
		377
		266
		241
		108
		102
Loss of tooth	2	119
		88
Replacement with a permanent crown	7	237
		98
		98
		98
		45
		18
		18

Overall, the survival rate of the 3D printed temporary crowns was 98% at the end of the observation period, thus the null hypothesis had to be rejected.

### Clinical parameters

**Figure 3:**
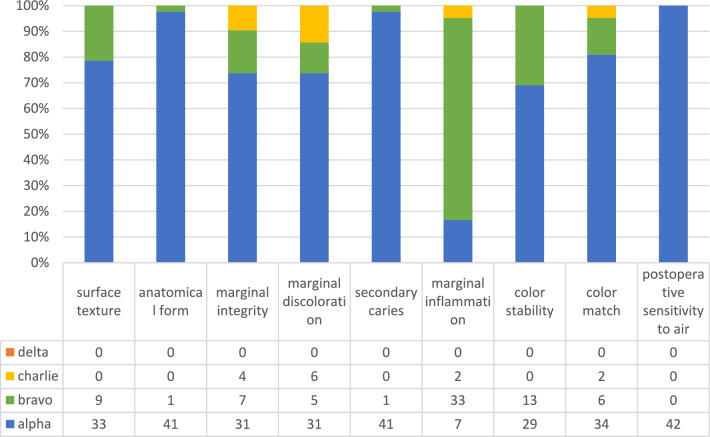
Bar chart of modified USPHS criteria. (Source: own figure).

As further analysis was conducted for 42 temporary crowns of 24 patients, their mean observation time (standard deviation) was 210 (± 120) days, reaching from 29 to 535 days (Table 2). Upon analysis with the Kolmogorov Smirnov test, no normality of the distribution of USPHS data was observed (*p* < 0.001). Data was visualized with a bar chart (Fig. 3).

The following three aspects were evaluated ≥ 98% with “alpha”: anatomical form, secondary caries and postoperative sensitivity to air. “Alpha” was scored at 81% for color match, 79% for surface texture, 74% for both marginal integrity and marginal discoloration and 69% for restoration color stability. Marginal inflammation was evaluated at 79% with “bravo”.

### Patient satisfaction with aesthetics

For the cohort including 42 temporary crowns of 24 patients, data was analyzed for the baseline session (T0) regarding satisfaction with aesthetics in the corresponding area, and for the recall sessions (T1) regarding satisfaction with aesthetics of the temporary crown. A Kolmogorov Smirnov test revealed no normality of the distribution of data (T0 *p* = 0.016, T1 *p* < 0.001).

**Figure 4:**
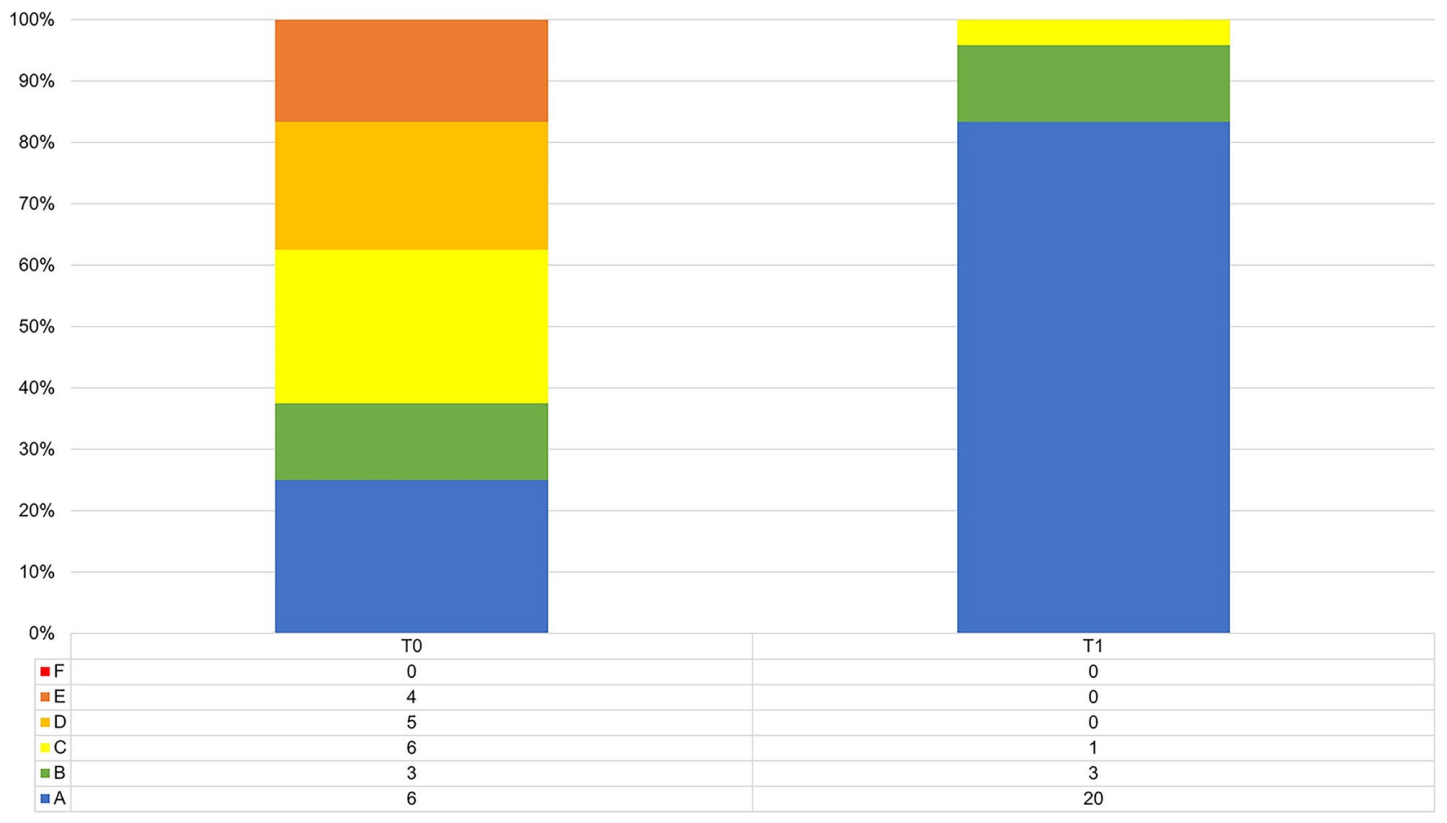
Bar chart for patient satisfaction with aesthetics for T0 and T1 (rated with the Wong-Baker FACES scale, “A” representing the happiest face and “F” the saddest face). (Source: own figure).

83% of patients rated their satisfaction with aesthetics with the happiest face at T1, compared to 25% at T0. Overall, patient satisfaction with aesthetics improved from T0 to T1 significantly (*p* < 0.001). Data is visualized in a bar chart (Fig. 4).

### Impact on OHRQoL

For 42 restorations of 24 patients, OHIP 14 data was analyzed for the baseline (T0) and at recall (T1) for evaluation of an impact on OHRQoL. OHIP mean sum values (standard deviation) were 6.63 (± 6.80) for T0 and 2.21 (± 4.53) for T1. Upon a Kolmogorov Smirnov test, the data was seen to not be normally distributed (T0 and T1 *p* < 0.001). Cronbach’s alpha was α = 0.788 for T0 and α = 0.803 for T1, yielding good reliability (Table 4). The Wilcoxon test revealed a significant (*p* = 0.005) improvement in OHIP sum values from T0 to T1, implying a significantly positive impact on OHRQoL.

**Table 4:** OHIP 14 sum values for T0 and T1.

	T0	T1
Mean	6.63	2.21
Standard deviation	6.80	4.53
Minimum	0	0
Maximum	23	19
Cronbach’s alpha	0.788	0.803

## Discussion

The study included 98 temporary crowns of 63 patients for survival analysis, with a mean observation time of 266 days, and further analysis for 42 restorations of 24 patients, with a mean observation time of 210 days. Observation time and restorations were limited, however, Permanent Crown Resin was released in 2020 as a limiting factor overall. Studies with s-CAM resin based restorations had observation periods of several years, e.g., 5 years^4^, and data on conventionally fabricated crowns exists for longer observation periods, e.g., 15 years^8^. As this study dealt with temporary crowns, although cemented permanently with Rely X Unicem, the observation period seems to be appropriate.

As there is an increasing trend in the accessibility and growth of the 3D printing industry^9^, several materials for the fabrication of permanent fixed dental prothesis have been introduced^10^. The Form3B 3D printer is equipped with a 250mW laser and features an XY resolution of 25µm and a layer thickness of 25µm, varying by material. CAD/CAM allows fast and reproducible production processes^11^, enabling an easy reproduction in case of a failure. A single crown requires approximately 0.3ml of resin, resulting in approximately 0.40€ costs—thus representing a feasible production.

The findings of this study revealed a survival rate of 98% with two catastrophic failures such that at the end of the observation rate 96 restorations remained in-situ. Thus, it could be stated that the 3D printed temporary crowns performed equally to s-CAD resin based restorations, based upon data from other studies with a survival of 100% after 6 months^5^, and the null-hypothesis was rejected.

3D printed crowns can be utilized as temporary crowns in pre-prosthetics, as in this study, or for testing new vertical dimensions in complex cases^12^. The findings do not underline the necessity of a temporization and there was no resulting interference with the restorations’ success, prognosis or demise. USPHS criteria are often used for evaluation of dental restorations, they have been criticized and FDI criteria were recommended^13,14^. The evaluation of the restorations’ clinical parameters was conducted in preclinical education, supervised by experienced dentists. Although calibration of examiners is recommended^15^, this could not be taken into account within this study’s design.

Permanent Crown Resin is a ceramic infiltrated resin, however, surface texture was evaluated as “bravo” for 21%. Several studies showed, that aging processes have an influence on the surface structure of composite resins^16,17^ and Papathanasiou et al. additionally found visible but acceptable color changes for milled composite resins. Thus, the results of the present study are in line with these findings. Marginal inflammation was evaluated at 79% with “bravo”, however, teeth were partially in compromitted state, yielding the indication of a temporary crown. This might explain the relatively high rate of inflammation in these compromised teeth. Although no other in vivo or in vitro studies exist with Permanent Crown Resin to the authors’ knowledge, composites are compatible with subgingival health^18^. Wuersching et al. concluded that monomer composition, presence and type of photo initiator and mode of polymerization affected the biocompatibility of dental resins and suggested further post-processing, i.e. additional light curing and washing, to improve the biocompatibility^10^. OHIP 14 sum values revealed a significant improvement (*p* = 0.005) from T0 to T1, implying a significant positive impact on OHRQoL. OHIP 5, the shortened form with 5 items, can already provide an insight into patients’ perception^19^. Overall, the cohort was very inhomogeneous and could not be stratified in discrete subgroups due to the limited number of patients. However, factors such as position of the restoration, age and gender of the patients affect the OHRQoL^20,21^.

A significant improvement was found regarding patient satisfaction with aesthetics. However, the cohort was very inhomogeneous, and restorations were located at various regions. Thus, generalization of the findings is limited. The Wong-Baker FACES scale is an established instrument^22^, whereas visual analog scales provide an objective and reliable instrument with high validity^23^.

The 3D printed temporary crowns were monolithic with a homogenous color. Advanced aesthetics could be achieved by utilizing light-cured glaze and characterization, such as GC OPTIGLAZE™ (GC Cooperation, Hongo, Bunkyo-ku, Tokyo).

Patient related variables such as parafunctional habits, caries activity, general and periodontal health, as well as operator related variables influence can influence dental restorations’ survival^24^. The absence of exclusion criteria, resulting in inclusion of teeth with diverse conditions before treatment and inclusion of patients with diverse risk factors, and diverse operators represent limitations of the study’s findings. Future studies should contain further stratification to analyze different risk factors individually upon the restorations’ survival and clinical parameters.

## Conclusions

The 3D printed temporary crowns fabricated out of Permanent Crown Resin achieved a survival rate of 98% within this retrospective cohort study. Patients benefitted from significant improvements in oral health-related quality of life and satisfaction with aesthetics. The analysis of clinical parameters demonstrated encouraging results of the 3D printed temporary crowns.

## Data Availability

All data needed to evaluate the conclusions in the paper are present in the paper and/or the Supplementary Materials.
